# Regioselective Benzoylation of Diols and Carbohydrates by Catalytic Amounts of Organobase

**DOI:** 10.3390/molecules21050641

**Published:** 2016-05-17

**Authors:** Yuchao Lu, Chenxi Hou, Jingli Ren, Xiaoting Xin, Hengfu Xu, Yuxin Pei, Hai Dong, Zhichao Pei

**Affiliations:** 1 Shaanxi Key Laboratory of Natural Products & Chemical Biology, College of Science, Northwest A & F University, Yangling 712100, China; luyuchao_2006@126.com (Y.L.); houchenxi2015@126.com (C.H.); renjingli12345@163.com (J.R.); 18700814753@163.com (X.X.); xhf130@126.com (H.X.); peiyx@nwafu.edu.cn (Y.P.); 2 School of Chemistry & Chemical Engineering, Huazhong University of Science & Technology, Luoyu Road 1037, Wuhan 430074, China

**Keywords:** benzoylation, regioselectivity, DBU, benzoylimidazole, catalysis

## Abstract

A novel metal-free organobase-catalyzed regioselective benzoylation of diols and carbohydrates has been developed. Treatment of diol and carbohydrate substrates with 1.1 equiv. of 1-benzoylimidazole and 0.2 equiv. of 1,8-diazabicyclo[5.4.0]undec-7-ene (DBU) in MeCN under mild conditions resulted in highly regioselective benzoylation for the primary hydroxyl group. Importantly, compared to most commonly used protecting bulky groups for primary hydroxyl groups, the benzoyl protective group offers a new protection strategy.

## 1. Introduction

**Scheme 1:**
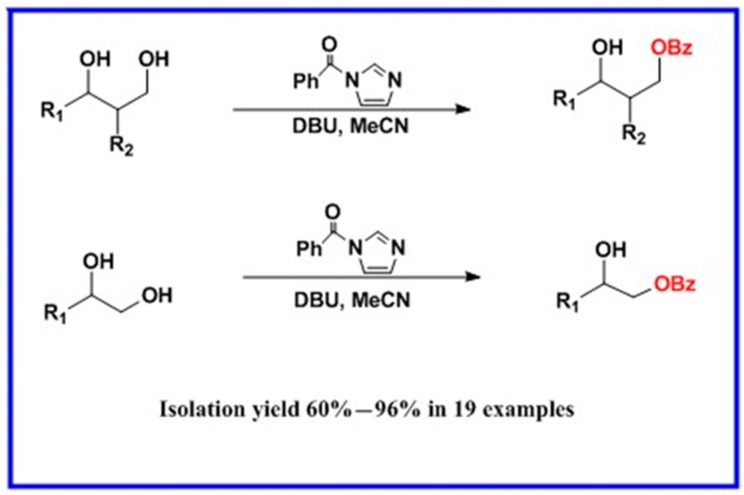
DBU-catalyzed benzoylation of 1,2 and 1,3-diols.

Selective protection of diols and polyols remains as a frequently encountered problem in organic chemistry during the synthesis of natural products and new drug candidates, especially, in carbohydrate chemistry [1,2,3,4]. Regioselectivity is the major obstacle to the synthesis of novel medicinal agents from readily available monosaccharide derivatives [5,6,7,8]. More specifically, developing environment-friendly, convenient, efficient and highly regioselective carbohydrate protection methods remains as one of the most prominent challenges [9]. Over the last several decades, many protection methods have been developed, including the use of reagents such as organotin [10,11,12], organoboron [13,14], organosilicon [15,16,17], metal salts [18,19,20,21,22,23], organobase [24,25,26], and enzymes [27,28,29,30]. Although these reagents each have advantages, they also possess troublesome shortcomings including inherent toxicity, high cost and the necessity to pre-protect secondary hydroxyl groups. One of the most frequently used protection methods is benzoylation. Many regioselective benzoylation methods using the above discussed reagents [20,21,22,25,26,27], have been reported accordingly. The Mitsunobu reaction is widely used in substitution of primary or secondary alcohols with nucleophiles mediated by a redox combination of a trialkyl or triarylphosphine and a dialkyl azodicarboxylate [31]. It may lead to regioselective benzoylation in some cases, but has poor regioselectivity and is more suitable for protection of secondary hydroxyls [32]. The DCC-mediated reaction, popularly known as the Steglich Esterification, may also lead to moderate regioselectivities in the benzoylation of hydroxyl groups with an excess amount of alcohol [33]. Albert and co-wokers used DMAP and DCC as counterion to obtain direct regioselective acetylation of octyl β-d-glucopyranoside, but with unsatisfactory results [34]. Recently, a green regioselective benzoylation method using benzoate anion as a catalyst was developed by us [35]. However, the method is not suitable for monobenzoylation of the unprotected methyl d-pyranosides. We also developed a regioselective acetylation method conducted in water with tetramethyl-ammonium hydroxide (TMAH) as a catalyst [36]. Despite its high selectivity, its main drawback is that it cannot be applied on substrates with poor water solubility. Thus, we started to investigate organobase-(DBU)-catalyzed selective benzoylation of diols and carbohydrates in organic solvents such as acetonitrile, where 1-benzoylimidazole was used as the acylation reagent, as in our previous study. As expected, this method showed good regioselectivity for the primary hydroxyl group in most cases (Scheme 1). Compared to other methods, not only does this robust method show better regioselectivities, but the catalyst is metal-free, and the reagents used are of lower cost, and easily acquired. Particularly, in contrast to the most often used protecting bulky groups for primary hydroxyl groups such as silyl [37] or trityl groups [38] which are removed under acid conditions, removal of the benzoyl group can take place in the presence of base. 

**Scheme 2:**
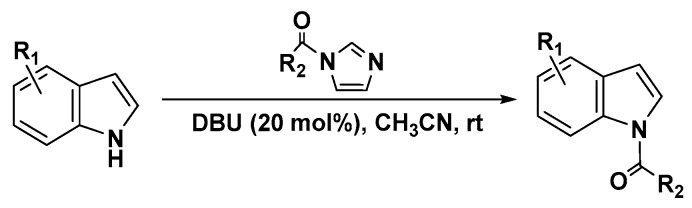
*N*-acetylation of heterocycles with carbonyl-azoles.

The carbonylimidazole derivatives are a class of highly acylation reagents, particularly in the preparation of esters and amides [39,40,41,42]. In several cases, they have shown superior selectivity for selective acylation reactions [43,44,45] and *N*-acetylation of oxindoles and indoles (Scheme 2) [46]. Inspired by these works, we decided to explore the possibility of replacing the acyl halide, which is toxic and unstable, with 1-benzoylimidazole as the benzoylation reagent with 1,8-diazabicyclo-[5.4.0]undec-7-ene (DBU) as the catalyst. DBU is often used as a organobase catalyst in many base-catalyzed reactions [47,48,49,50], having been recognized as an excellent nucleophilic catalyst, leading to rapid and efficient acetylation of non-nucleophilic azacycles, such as indoles, pyrroles, and oxazolidinones.

## 2. Results and Discussion

After a series of optimization studies on selective benzoylation of methyl α-d-glucopyranoside (**1**, Table 1), we determined the best condition to be the use of 0.2 equiv. of DBU and 1.1 equiv. of 1-benzoylimidazole in acetonitrile with a small amount of DMF (MeCN/DMF: 20/1) at 50 °C for 8 h (Entry 1). Under this condition, methyl glucoside **2** with a benzoylated primary hydroxyl group was isolated in 70% yield. The yield of **2** was only increased to 72% when 1.3 equiv. of 1-benzoylimidazole was used (Entry 2). Without the addition of DMF the yield decreased to 53%, which is likely due to the low solubility of polyols in pure acetonitrile (Entry 3). If the reaction was performed at room temperature, the yield of **2** decreased to only 21% (Entry 4). Increasing the reaction temperature to 70 °C or prolonging the reaction time to 12 h. failed to improve the yield of **2** (Entries 5–6). No reaction occurred in the absence of DBU or with Et_3_N instead of DBU (Entries 7–8). Low yields were obtained with DMAP, DIPEA or DBN instead of DBU (Entries 9, 10 and 11). The yield of **2** increased with the amount of DBU used up to 0.4 equiv (Entries 12, 13 and 14).

In light of these results, a wide range of substrates was further selectively benzoylated under the optimized conditions (Table 2), including 1,2-diols **3**, **5**, **7**, **9**, **11** and **13** (Entries 1–6), 1,3-diols **15**, **17** and **19** (Entries 7–9), methyl d-pyranoside 1,3-diols **23**, **25**, **27** and **29** (Entries 11–14), and carbohydrate polyols **31**, **33**, **35** and **37** (Entries 15–18). The addition of DMF is not necessary for diols due to their good solubility in acetonitrile. All of the substrates contain one primary hydroxyl group with one or several secondary hydroxyl groups. Good yields (60%–96%) where the primary hydroxyl group was selective protected were obtained in most cases. For non-carbohydrate diols, the only by-products were a small amount of overbenzoylated compounds (Entries 1–7). Particularly, excellent selectivities (87%–96%) were shown due to striking steric effects for the benzoylation of 1,3-diols **17** and **19** (Entries 8 and 9), containing a tertiary hydroxyl group, and the five carbohydrate diols **21**, **23**, **25**, **27** and **29** (Entries 10–14).

**Figure 1:**
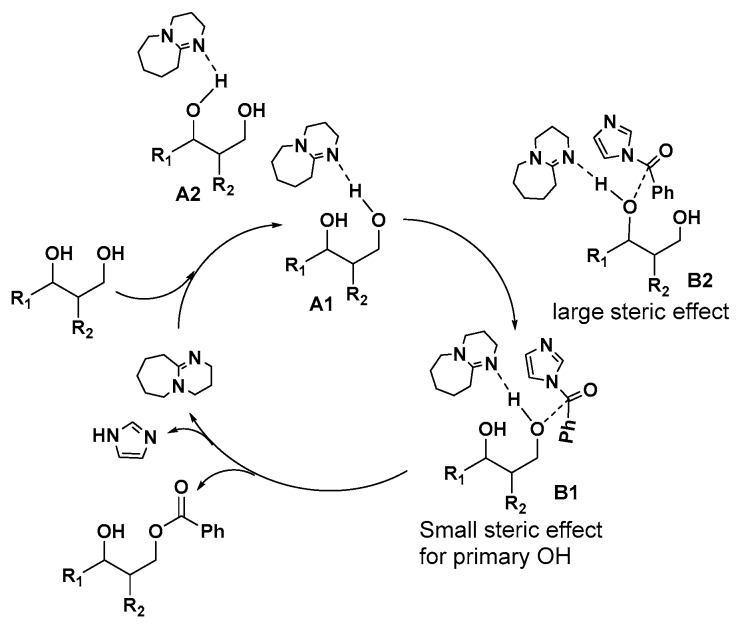
Proposed mechanism for selective benzoylation of primary OH using 1-benzoylimidazole as acylation reagent catalyzed by DBU.

Evidently, the benzoylation occcurs via by transacylation of imidazolides under basic condition [51,52,53]. Based on the traditional base-catalysis principle, this transacylation is usually proposed to start from a deprotonation of hydroxyl groups in the presence of strong base such as DBU. Recently, it was proposed that the hydrogen bond between the hydroxyl group and the base play a key role in the transacylation reaction [54,55,56]. Thus, a mechanism based on hydrogen bond principle for this selective benzoylation is proposed in Figure 1. H-bond complexes **A1** and **A2** can be formed between the hydroxyl group of substrate and DBU, following the formation of the transition state intermediates **B1** and **B2** with the approach of 1-benzoylimidazole. It is clear that the formation of intermediate **B1** is more favoured due to the less steric hindrance of **A1**. In the transition state (TS) **B**, the deprotonation of the hydroxyl group by DBU and the attack of the hydroxyl group on 1-Benzoylimidazole occur at the same time. Consequently, the markedly less steric hindrance in **A1** and **B1** than in **A2** and **B2** explained the good selectivity for benzoylation of the primary hydroxyl group.

## 3. Materials and Methods 

### 3.1. General Information

All commercially available starting materials and solvents were of reagent grade and dried prior to use. Chemical reactions were monitored with thin-layer chromatography using precoated silica gel 60 (0.25 mm thickness) plates. Flash column chromatography was performed on silica gel 60 (0.040–0.063 mm). ^1^H- and ^13^C-NMR spectra were recorded with a Bruker AVANCE III instrument operating at 500 and 125 MHz, and using the residual signals from DMSO-*d*_6_ (^1^H: δ = 2.50 ppm), CD_3_OD (^1^H: δ = 3.34 ppm) and CDCl_3_ (^1^H: δ = 7.27 ppm) as internal standard.

### 3.2. General Procedure for Selective Benzoylation of Diols (a)

To a solution of substrate (100 mg) in 2.5 mL MeCN (dry) was added DBU (0.2 equiv.), and the mixture was allowed to stir at 50 °C for 10 min. 1-Benzoylimidazole (1.1 equiv.) in MeCN (dry, 0.5 mL) was added to the reaction mixture in two portions and it was allowed to stir at 50 °C for 8 h. MeCN was removed under reduced pressure and the resulting mixture was purified by flash column chromatography (ethyl acetate/petroleum ether = 1:6 to 2:1) to afford benzoylated products.

### 3.3. General Procedure for Selective Benzoylation of Polyols (b)

To a solution of substrate (100 mg) in 2.5 mL MeCN (dry) and 0.15 mL DMF (dry) was added DBU (0.2 equiv.), and the mixture was allowed to stir at 50 °C for 10 min. 1-Benzoylimidazole (1.1 equiv.) in 0.5 mL MeCN (dry) was added to the reaction mixture in two portions and it was allowed to stir at 50 °C for 8 h. MeCN was removed under reduced pressure and the resulting mixture was purified by flash column chromatography (ethyl acetate/petroleum ether = 1:1 to 10:1) to afford benzoylated products.

### 3.4 Product Characterization

*3-(Allyloxy)propane-1,2-diyl dibenzoate* (**8b**). Colorless oil, yield: 76%, Rf = 0.2 (ethyl acetate/petroleum ether = 1:3), ^1^H-NMR (CDCl_3_) δ 8.09–7.99 (m, 4H, Ar*H*), 7.59–7.52 (m, 2H, Ar*H*), 7.47–7.39 (m, 4H, Ar*H*), 5.94–5.83 (m, 1H, -C*H*=CH_2_), 5.65–5.56 (m, 1H, -C*H*-), 5.33–5.15 (m, 2H, -CH=C*H*_2_), 4.72–4.58 (m, 2H, -C*H*_2_COOPh), 4.09–4.04 (m, 2H, -C*H*_2_CH=CH_2_), 3.84–3.76 (m, 2H, -C*H*_2_OCH_2_CH=CH_2_). ^13^C-NMR (CDCl_3_) δ 166.7, 166.3, 150.1, 148.0, 133.2, 129.8, 128.5, 122.6, 121.1, 115.8, 115.5, 112.1, 71.6, 68.6, 65.7, 55.9. HRMS *m*/*z* calcd for C_20_H_20_O_5_ [M + Na]^+^: 363.1203. Found 363.1207.

*2-Hydroxy-3-(2-methoxyphenoxy)propyl benzoate* (**12a**). Colorless oil, yield: 75%, Rf = 0.2 (ethyl acetate/petroleum ether = 1:2), ^1^H-NMR (CDCl_3_) δ 8.12–7.22 (m, 2H, Ar*H*), 7.64–7.58 (m, 1H, Ar*H*), 7.51–7.45 (m, 2H, Ar*H*), 7.07–6.99 (m, 2H, Ar*H*), 6.98–6.91 (m, 2H, Ar*H*), 4.61–4.51 (m, 2H, PhCO_2_C*H*_2_-), 4.46–4.38 (m, 1H, -*H*COH-), 4.28–4.12 (m, 2H, -PhOC*H*_2_-), 3.90 (s, 3H, -OC*H*_3_). ^13^C-NMR (CDCl_3_) δ 165.9, 150.3, 148.0, 133.3, 133.2, 129.9, 128.5, 122.6, 121.0, 115.8, 112.5, 70.7, 68.2, 63.4, 55.9. HRMS *m*/*z* calcd for C_17_H_18_O_5_ [M + Na]^+^: 325.1046. Found 325.1051.

*3-(2-Methoxyphenoxy)propane-1,2-diyl dibenzoate* (**12b**). Colorless oil, yield: 15%, Rf = 0.2 (ethyl acetate/petroleum ether = 1:3), ^1^H-NMR (CDCl_3_) δ 8.11–8.05 (m, 4H, Ar*H*), 7.63–7.57 (m, 2H, Ar*H*), 7.50–7.43 (m, 4H, Ar*H*), 7.09–6.99 (m, 2H, Ar*H*), 6.96–6.93 (m, 2H, Ar*H*), 5.87–5.80 (m, 1H, -CH_2_C*H*CH_2_-),δ 4.88 (dd, *J* = 12.0, 3.8 Hz, 1H, PhCO_2_C*H*_2_-), 4.80 (dd, *J* = 12.0, 6.0 Hz, 1H, PhCO_2_C*H*_2_-), 4.46 (d, *J* = 5.4 Hz, 2H, -PhOC*H*_2_-), 3.84 (s, 3H, -OCH_3_). ^13^C-NMR (CDCl_3_) δ 166.3, 165.9, 150.2, 148.0, 134.3, 133.3, 133.2, 133.1, 129.8, 129.7, 128.5, 128.4, 122.7, 121.1, 117.5, 112.1, 71.1, 68.3, 63.6, 55.9. HRMS *m*/*z* calcd for C_24_H_22_O_6_ [M + Na]^+^: 429.1309. Found 429.1312.

## 4. Conclusions 

In conclusion, a simple and efficient method for regioselective benzoylation of diols and carbohydrates has been developed, where the primary hydroxyl groups can be selectively benzoylated by using 1-benzoylimidazole as an acylation reagent catalyzed by DBU. In comparison to other methods, not only does this robust method appear to have provided a new protection strategy for the primary hydroxyl group, but also the used reagents are associated with lower cost, metal-free, and displayed better regioselectivity.

## Figures and Tables

**Table 1:** Optimization studies on selective benzoylation of methyl α-d-glucopyranoside (**1**) ^a^. 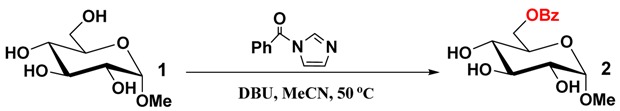

Entry 1	Various Conditions	Yield ^b^ (%)
1	Optimized conditions	70 (80 ^c^)
2	Benzoylimidazole (1.3 eq.)	72 (87 ^c^)
3	Without DMF	53
4	Reaction at r.t.	21
5	Reaction at 70 °C	69
6	Reaction for 12 h.	71
7	Without DBU	- ^d^
8	Et_3_N (0.2 eq.) instead of DBU	- ^d^
9	DMAP (0.2 eq.) instead of DBU	18
10	DIPEA (0.2 eq.) instead of DBU	13
11	DBN (0.2 eq.) instead of DBU	45
12	DBU (0.05 eq.)	52
13	DBU (0.1 eq.)	61
14	DBU (0.4 eq.)	71

^a^ Conditions: methyl α-d-glucopyranoside (**1**, 100 mg), catalyst (0.2 eq.), 1-benzoylimidazole (1.1 eq.), MeCN (3 mL, dry), DMF (0.15 mL, dry), 50 °C, 8 h; ^b^ Isolated yield; ^c^ Yields determined by ^1^H-NMR; ^d^ No reaction; DBU: 1,8-diazabicyclo[5.4.0]undec-7-ene, DMAP: 4-dimethylaminopyridine, DBN: 1,5-diazabicyclo[4.3.0]non-5-ene.

**Table 2:** 1-Benzoylimidazole used for selective benzoylation of diols and carbohydrates.

Entry	Reactant	Products	Isolated Yield ^a^ (%)
1	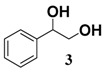	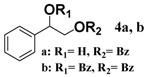	a/b(82/8)
2	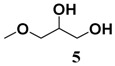	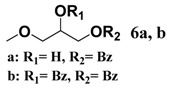	a/b(75/12)
3	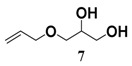	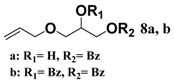	a/b(76/11)
4	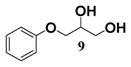	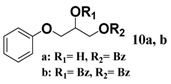	a/b(81/10)
5	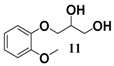	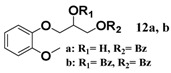	a/b(75/15)
6	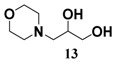	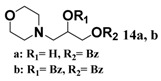	a/b(76/9)
7	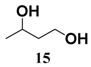	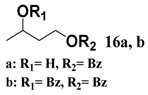	a/b(78/14)
8	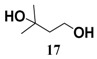	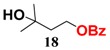	90
9	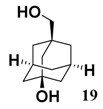	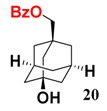	96
10	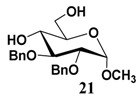	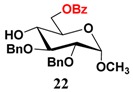	92
11	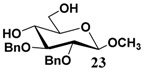	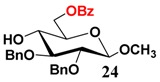	90
12	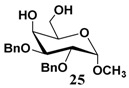	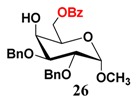	88
13	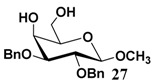	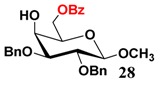	91
14	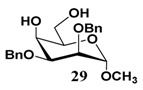	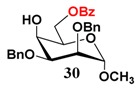	87
15	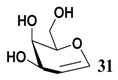	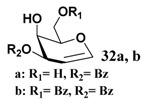	a/b(81/9)
16	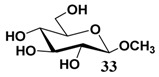	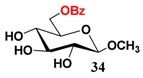	60 ^b^ (70 ^c^)
17	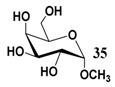	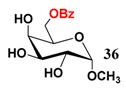	62 ^b^ (70 ^c^)
18	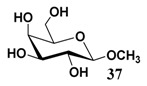	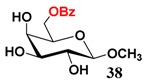	71 ^b^ (80 ^c^)

^a^ *Reaction conditions*: 1-benzoylimidazole (1.1 equiv.), DBU (0.2 equiv.), MeCN (3 mL, dry); ^b^ MeCN (3 mL, dry), DMF (0.15 mL, dry); ^c^ Yields determined by ^1^H-NMR.

## Supplementary Materials

Supplementary materials can be accessed at: http://www.mdpi.com/1420-3049/21/5/641/s1.
