# Supplementary material for: Regioselective Benzoylation of Diols and Carbohydrates by Catalytic Amounts of Organobase
Source: Molecules. 2016 May 17;21(5):641. doi: 10.3390/molecules21050641 (PMC6274181; doi:10.3390/molecules21050641)
Supplement: Supplementary file 1 [file molecules-21-00641-s001.pdf]

Supplementary Materials: Regioselective Benzoylation of Diols and Carbohydrates by Catalytic Amounts of Organobase

Yuchao Lu, Chenxi Hou, Jingli Ren, Xiaoting Xin, Hengfu Xu, Yuxin Pei, Hai Dong and Zhichao Pei

Contents

1. Characterization of Important Known Compounds ---S2

2. ^{1}H-NMR and ^{13}C-NMR Spectra ---S4

Reference---S7

1. Characterization of Important Known Compounds

Methyl 6-O-benzoyl-α-D-glucopyranoside (**2**): ^{1}H-NMR (500 MHz, $CDCl_3$) δ 8.05–7.98 (m, 2H), 7.53–7.50 (m, 1H), 7.39 (m, 2H), 4.78 (d, *J* = 3.4 Hz, 1H, H_1), 4.67 (dd, *J* = 12.1, 4.8 Hz, 1H), 4.54 (d, *J* = 11.8 Hz, 1H), 3.86 (dd, *J* = 9.6, 3.2 Hz, 2H), 3.80 (t, *J* = 8.9 Hz, 1H), 3.56 (d, *J* = 6.2 Hz, 1H), 3.48 (d, *J* = 9.5 Hz, 1H), 3.43 (s, 3H, OMe) [1].

2-Hydroxy-2-phenylethyl benzoate (**4a**): ^{1}H-NMR (500 MHz, $CDCl_3$) δ 8.11–8.03 (m, 2H), 7.64–7.54 (m, 1H), 7.45 (dd, *J* = 10.7, 4.7 Hz, 4H), 7.41–7.35 (m, 3H), 5.12 (d, *J* = 8.1 Hz, 1H), 4.54 (dd, *J* = 11.6, 3.5 Hz, 1H), 4.43 (dd, *J* = 11.6, 8.2 Hz, 1H) [2].

1-Phenylethane-1,2-diyl dibenzoate (**4b**): ^{1}H-NMR (500 MHz, $CDCl_3$) δ 8.19–8.12 (m, 2H), 8.08–8.00 (m, 2H), 7.66–7.55 (m,4H), 7.52–7.36 (m, 7H), 6.47 (dd, *J* = 8.2, 3.7 Hz, 1H), 4.80 (dd, *J* = 11.9, 8.2 Hz, 1H), 4.72 (dd, *J* = 11.9, 3.7 Hz, 1H) [3].

2-Hydroxy-3-methoxypropyl benzoate (**6a**): ^{1}H-NMR (500 MHz, $CDCl_3$): δ 8.09–8.03 (m, 2H, Ph), 7.60–7.55 (m, 1H, Ph), 7.48–7.41 (m, 2H, Ph), 4.45–4.36 (m, 2H, CH_2OCOPh), 4.19–4.12 (m, 1H, C*H*OH), 3.59–3.47 (m, 2H, CH_2OCH_3), 3.42 (s, 3H, OCH_3) [4].

3-Methoxypropane-1,2-diyl dibenzoate (**6b**): ^{1}H-NMR (500 MHz,$CDCl_3$) δ 8.13–8.04 (m, 4H, Ph), 7.64–7.57 (m, 2H, Ph), 7.51–7.44 (m, 4H, Ph), 5.68–5.62 (m, 1H, C*H*OCOPh), 4.74–4.62 (m,2H, CH_2OCOPh), 3.84–3.76 (m, 2H, CH_2OCH_3), 3.48 (s, 3H, OCH_3) [4].

3-(Allyloxy)-2-hydroxypropyl benzoate (**8a**): ^{1}H-NMR (500 MHz, $CDCl_3$) δ 8.3–8.07 (m, 2H, Ph), 7.64–7.59 (m, 1H, Ph), 7.53–7.45 (m, 2H, Ph), 6.01–5.90 (m, 1H), 5.37–5.22 (m, 2H), 4.52–4.41 (m, 2H), 4.27–4.18 (m, 1H), 4.15–4.07 (m, 2H), 3.63 (ddd, *J* = 15.8, 9.7, 5.2 Hz, 2H) [3].

3-Phenoxypropyl benzoate (**10a**): ^{1}H-NMR (500 MHz, $CDCl_3$) δ 8.17–8.06 (m, 2H), 7.69–7.59 (m, 1H), 7.56–7.45 (m, 2H), 7.40–7.32 (m, 2H), 7.10–7.00 (m, 1H), 7.02–6.92 (m, 2H), 4.67–4.55 (m, 2H), 4.48–4.40 (m, 1H), 4.17 (qd, *J* = 9.5, 5.2 Hz, 2H) [5].

3-Phenoxypropane-1,2-diyl dibenzoate (**10b**): ^{1}H-NMR (500 MHz, $CDCl_3$) δ 8.08 (dt, *J* = 12.2, 6.1 Hz, 4H), 7.61 (dd, *J* = 13.6, 7.3 Hz, 2H), 7.48 (dd, *J* = 13.8, 7.6 Hz, 4H), 7.40–7.32 (m, 2H), 7.09–6.95 (m, 3H), 5.88–5.78 (m, 1H), 4.92–4.75 (m, 2H), 4.48–4.33 (m, 2H) [5].

Morpholinopropyl benzoate (**14a**): ^{1}H-NMR (500 MHz, $CDCl_3$): δ 8.12–8.09 (m, 2H, Ar*H*), 7.64–7.58 (m, 1H, Ar*H*), 7.52–7.46 (m, 2H, Ar*H*), 4.46–4.33 (m, 2H, $PhCO_2CH_2$-), 4.17–4.11 (m, 1H, $PhCO_2CH_2CH$-),3.79–3.73 (m, 4H, -CH_2OCH_2-), 2.75–2.69 (m, 2H, -CH_2CH(OH)-), 2.55(m, 2H, -$CH_2CH_2OCH_2$-), 2.53–2.48 (m, 2H, -$CH_2OCH_2CH_2$-) [6].

3-Morpholinopropane-1,2-diyl dibenzoate (**14b**): ^{1}H-NMR (500 MHz, $CDCl_3$): δ 8.12–8.04 (m, 4H), 7.67–7.57 (m, 2H), 7.55–7.41 (m, 4H), 5.66 (qd, *J* = 6.4, 3.2 Hz, 1H), 4.75 (dd, *J* = 11.9, 3.2 Hz, 1H), 4.69–4.57 (m, 1H), 3.77–3.68 (m, 4H), 2.82–2.72 (m, 2H), 2.68–2.55 (m, 4H) [7].

3-Hydroxybutyl benzoate (**16a**): ^{1}H-NMR (500 MHz, $CDCl_3$): δ 8.11–8.05 (m, 2H, Ph), 7.64–7.56 (m, 1H, Ph), 7.52–7.45 (m, 2H, Ph), 4.74–4.37 (m, 1H), 3.78 (t, *J* = 77.9, 19.7, 7.1 Hz, 1H), 2.47–2.14 (m, 1H), 2.06–1.83 (m, 1H), 1.39 (dd, *J* = 73.3, 6.3 Hz, 2H) [3].

Butane-1,3-diyl dibenzoate (**16b**): ^{1}H-NMR (500 MHz, $CDCl_3$) δ 8.06–8.03 (m, 4H), 7.65–7.53 (m, 2H), 7.51–7.43 (m, 4H), 5.43 (dt, *J* = 12.7, 6.2 Hz, 1H), 4.62–4.44 (m, 2H), 2.31–2.13 (m, 2H), 1.49 (d, *J* = 6.3 Hz, 3H) [3].

3-Hydroxy-3-methylbutyl benzoate (**18**): ^{1}H-NMR (500 MHz, $CDCl_3$) δ 8.07 (m, 2H), 7.60 (t, *J* = 7.4 Hz, 1H), 7.48 (t, *J* = 7.7 Hz, 2H), 4.55 (t, *J* = 6.8 Hz, 2H), 2.03 (t, *J* = 6.8 Hz, 2H), 1.37 (s, 6H) [8].

3-Hydroxyadamantan-1-yl)methyl benzoate (**20**): ^{1}H-NMR ($CDCl_3$, 500 MHz): δ 8.07–8.02 (m, 2H, ph), 7.59–7.54 (m, 1H, Ph), 7.48–7.42 (m, 2H, Ph), 4.01 (s, 2H), 2.29–2.25 (m, 2H), 1.77–1.68 (m, 4H), 1.65–1.53 (m, 8H) [9].

Methyl 2,3-di-O-benzyl-6-O-benzoyl-α-D-glucopyranoside (**22**): ^{1}H-NMR (500 MHz, $CDCl_3$): δ 8.10–8.06 (m, 2H, Ph), 7.64–7.58 (m, 1H, Ph), 7.50–7.32 (m, 12H, Ph), 5.06 (d, 1H, *J* = 11.2 Hz, -C*H*$_2$Ph), 4.82 (m, 2H, -C*H*$_2$Ph), 4.76–4.64 (m, 3H, H-1, H-6), 4.59–4.53 (m, 1H, H-3), 3.97–3.85 (m, 2H, H-5,H-2), 3.61–3.54 (m, 1H, H-4), 3.45 (s, 3H, OCH_3) [10].

Methyl 2,3-di-O-benzyl-6-O-benzoyl-β-D-glucopyranoside (**24**): ^{1}H-NMR ($CDCl_3$, 500 MHz): δ 8.13–8.08 (m, 2H, Ph), 7.60–7.54(m, 1H, Ph), 7.49–7.28(m, 12H, Ph), 4.98–4.91 (m, 2H, -C*H*$_2$Ph), 4.76–4.69 (m, 2H, -C*H*$_2$Ph), 4.71–4.60 (m, 2H, H-6), 4.42 (d, *J* = 7.6 Hz, 1H, H-1), 3.66–3.63(m, 1H, H-4), 3.62 (s, 3H, OCH_3), 3.60–3.58 (m, 1H, H-3), 3.57–3.51 (m, 1H, H-2), 3.50–3.44 (m, 1H, H-5) [11].

Methyl 2,3-di-O-benzyl-6-O-benzoyl-α-D-galactopyranoside (**26**): ^{1}H-NMR ($CDCl_3$, 500 MHz): δ 8.09–8.05 (m, 2H, Ph), 7.64–7.58 (m, 1H, Ph), 7.51–7.31(m, 12H, Ph), 4.91–4.88(m, 1H, -C*H*$_2$Ph), 4.87 (d, *J* = 3.6 Hz, 1H, H-1), 4.79–4.70(m, 3H, -C*H*$_2$Ph), 4.63–4.51(m, 2H, H-6), 4.13–4.07(m, 2H, H-4,-H-5), 3.98–3.89 (m, 2H, H-2,H-3), 3.42 (s, 3H, OCH_3) [12].

Methyl 2,3-di-O-benzyl-6-O-benzoyl-β-D-galactopyranoside (**28**): ^{1}H-NMR ($CDCl_3$, 500 MHz): δ 8.06–8.02 (m, 2H, Ph), 7.57 (m, 1H, Ph), 7.47–7.27 (m, 12H, Ph), 4.90 (d, *J* = 11.1 Hz, 1H, -C*H*$_2$Ph), 4.78–4.68 (m, 3H, -C*H*$_2$Ph), 4.65–4.55 (m, 2H, H-6), 4.29 (d, *J* = 7.7 Hz, 1H, H-1), 3.99 (m, 1H, H-4), 3.76–3.72 (m, 1H, H-5), 3.68–3.62 (m,1H, H-2), 3.56 (s, 3H, OCH_3), 3.55–3.51 (m, 1H, H-3) [12].

Methyl 2,3-di-O-benzyl-6-O-benzoyl-α-D-mannopyranoside (**30**): ^{1}H-NMR ($CDCl_3$, 500 MHz): δ 8.14–8.06 (m, 2H, Ph), 7.63–7.55 (m, 1H, Ph), 7.49–7.30 (m, 12H, Ph), 4.88 (d, *J* = 2.9 Hz, 1H, *H-1*), 4.72–4.65 (m, 5H,-C*H*$_2$Ph, *H*-6), 4.58–4.54 (m, 1H, *H*-6), 4.19–4.12 (m, 1H, *H*-4), 3.94–3.85 (m, 2H, *H*-5, *H*-2, 3.82–3.78 (m, 1H, *H*-3), 3.42 (s, 3H, OC*H*$_3$) [13].

6-O-Benzoyl-D-galactal (**32a**): ^{1}H-NMR ($CDCl_3$, 500 MHz): δ 8.07–8.01 (m, 2H, Ph), 7.59–7.54 (m,1H, Ph), 7.47–7.41 (m,2H, Ph), 6.39 (dd, *J* = 6.4, 1.6Hz, 1H, *H*-1), 4.75–4.70 (m, 1H, *H*-2), 4.70–4.51 (m, 2H, *H*-6), 4.41 (m,1H, *H*-5), 4.22 (m, 1H, *H*-3), 4.00–3.93 (m, 1H, *H*-4) [14].

3,6-Di-O-benzoyl-D-galactal (**32b**): ^{1}H-NMR ($CDCl_3$, 500 MHz): δ 8.12–8.03 (m, 4H, Ph), 7.64–7.54 (m, 2H, Ph), 7.50–7.42 (m,4H, Ph), 6.57 (dd, *J* = 6.2, 1.5 Hz, 1H, *H-1*), 5.76–5.70 (m, 1H, *H-3*), 4.89–4.84 (m, 1H, *H*-2), 4.74–4.64 (m, 2H, *H-6*), 4.51–4.33 (m, 2H, *H*-5, *H-4*) [14].

Methyl 6-O-benzoyl-β-D-glucopyranoside (**34**): ^{1}H-NMR (500 MHz, $CDCl_3$): δ 8.05–7.98 (m, 2H), 7.53–7.50 (m, 1H), 7.39 (m, 2H), 4.67–4.62 (m, 1H), 4.56–4.51 (m, 1H), 4.22 (d, *J* = 7.7 Hz, 1H, H_1), 3.63–3.59 (m, 2H), 3.55–3.51 (m, 1H), 3.48 (s, 3H, OMe), 3.44–3.38 (m, 1H) [15].

Methyl 6-O-benzoyl-α-D-galactopyranoside (**36**): ^{1}H-NMR (400 MHz, $CDCl_3$): δ 8.08–8.03 (m, 2H), 7.59–7.55 (m, 1H), 7.47–7.43 (m, 2H), 4.72 (dd, *J* = 11.4, 6.8 Hz, 1H), 4.53 (dd, *J* = 11.3, 6.5 Hz, 1H), 4.21 (d, *J* = 7.4 Hz, 1H, H_1), 4.01–3.97 (m, 1H), 3.84 (d, *J* = 6.7 Hz, 1H), 3.67–3.64 (m, 2H), 3.57 (s, 3H, OMe) [16].

Methyl 6-O-benzoyl-β-D-galactopyranoside (**38**): ^{1}H-NMR (400 MHz, $CDCl_3$): δ 8.08–8.03 (m, 2H), 7.59–7.55 (m, 1H), 7.47–7.43 (m, 2H), 4.76 (dd, *J* = 11.2, 6.7 Hz, 1H), 4.56 (dd, *J* = 11.2, 6.6 Hz, 1H), 4.17 (d, *J* = 7.3 H, 1H, *H-1*), 3.84 (m, 2H), 3.69–3.65 (m, 2H), 3.37 (s, 3H, OMe) [16].

2. ^{1}H-NMR and ^{13}C-NMR Spectra

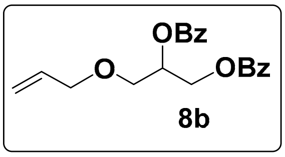

^{1}H-NMR
(500 MHz, $CDCl_3$)

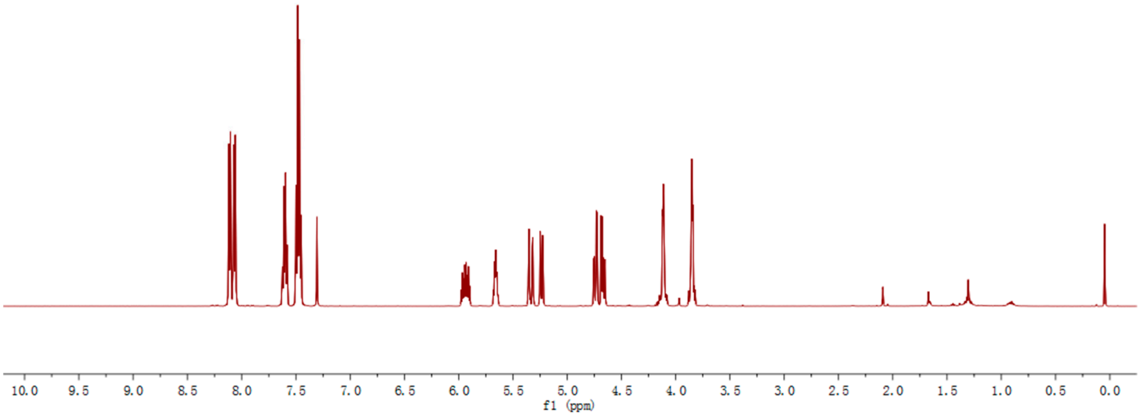

Figure S1. ^{1}H-NMR spectrum (500 MHz, $CDCl_3$) of **8b**.

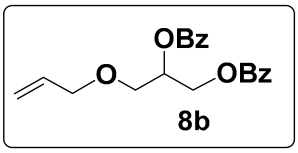

^{13}C-NMR
(125 MHz, $CDCl_3$)

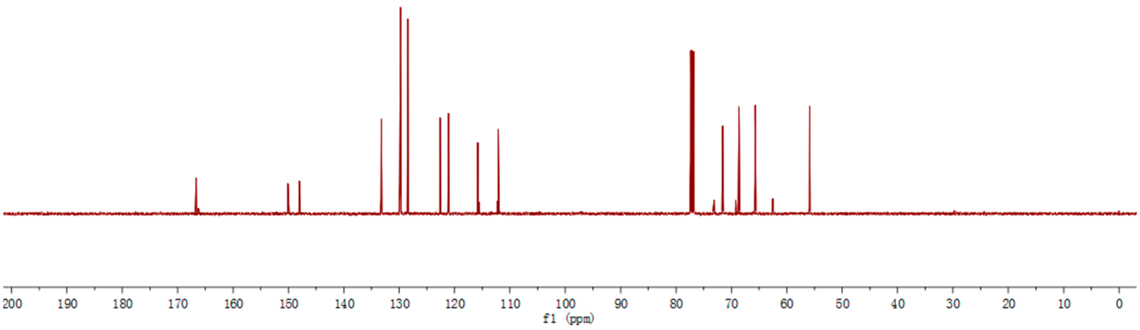

Figure S2. ^{13}C-NMR spectrum (125 MHz, $CDCl_3$) of **8b**.

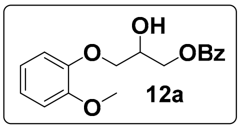

^{1}H-NMR
(500 MHz, $CDCl_3$)

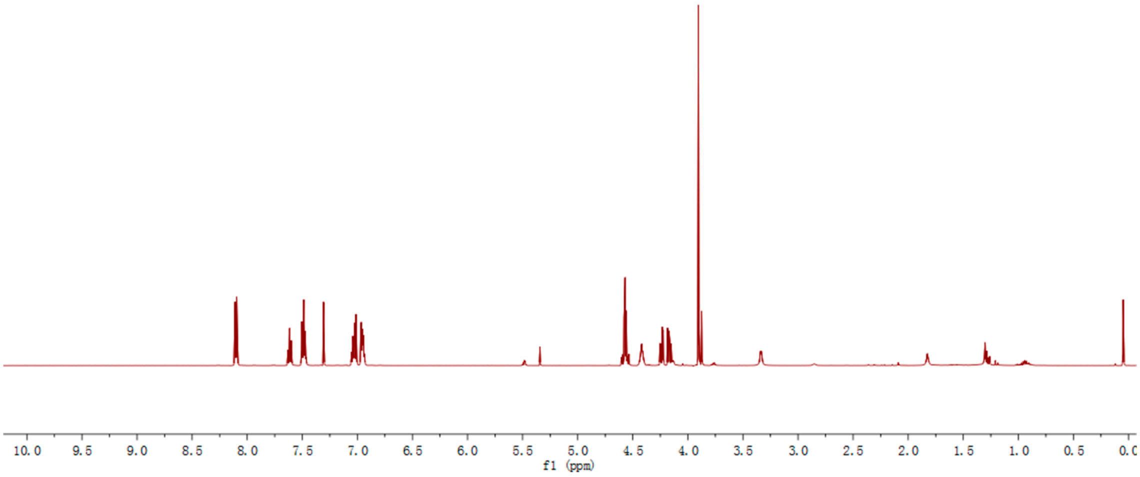

Figure S3. ^{1}H-NMR spectrum (500 MHz, $CDCl_3$) of **12a**.

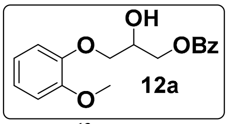

^{13}C-NMR
(125 MHz, $CDCl_3$)

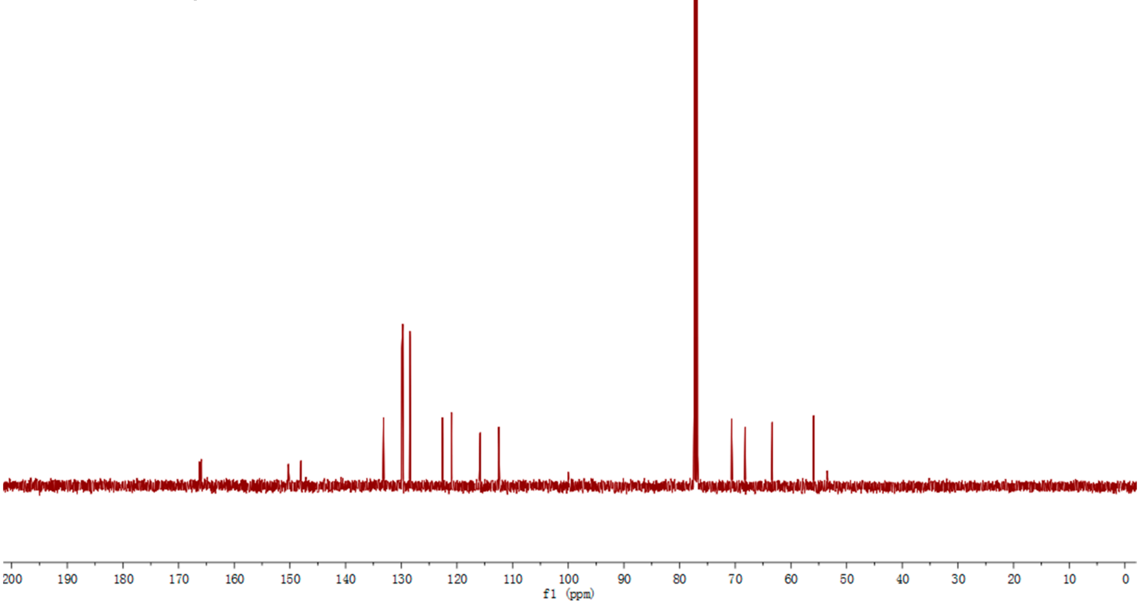

Figure S4. ^{13}C-NMR spectrum (125 MHz, $CDCl_3$) of **12a**.

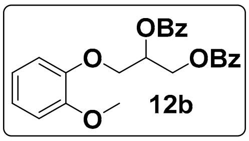

^{1}H-NMR
(500 MHz, $CDCl_3$)

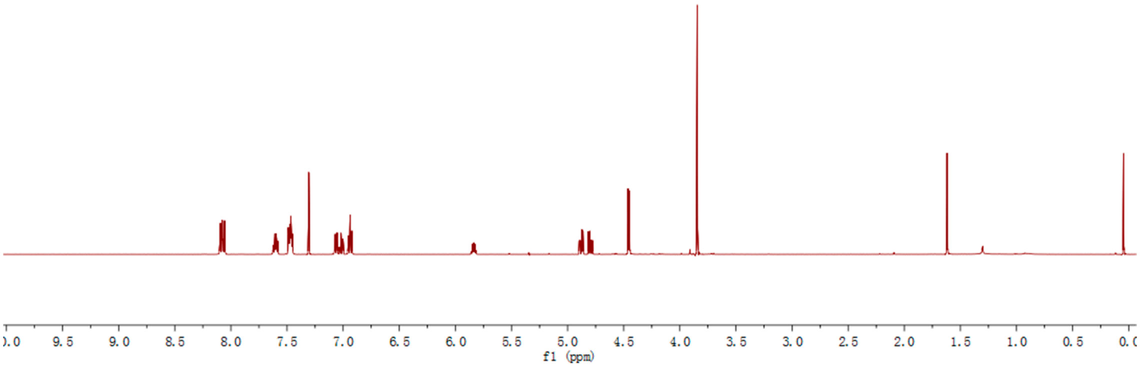

Figure S5. ^{1}H-NMR spectrum (500 MHz, $CDCl_3$) of **12b**.

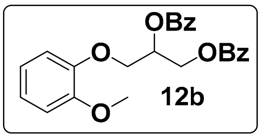

^{13}C-NMR
(125 MHz, $CDCl_3$)

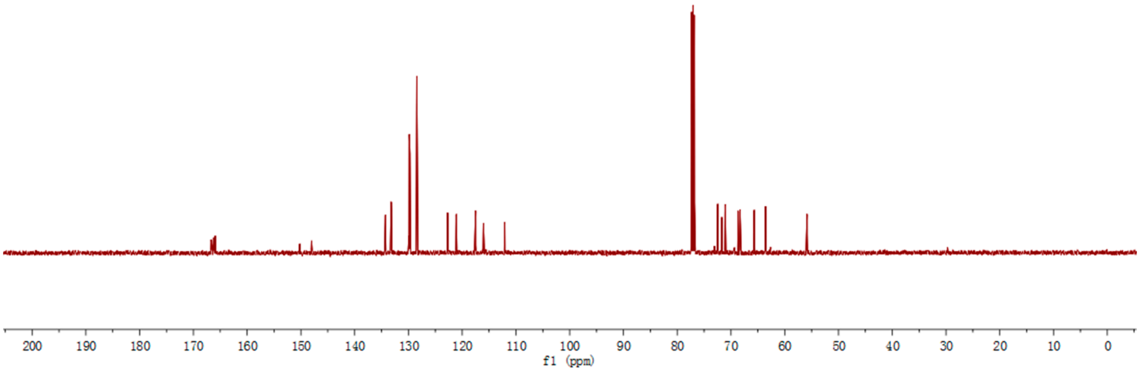

Figure S6. ^{13}C-NMR spectrum (125 MHz, $CDCl_3$) of **12b**.

Reference

1. Evtushenko, E.V. Regioselective benzoylation of glycopyranosides by benzoic anhydride in the presence of $Cu(CF_3COO)_2$. *Carbohydr. Res.* **2012**, *359*, 111–119.
2. Lee, D.; Williamson, C.L.; Chan, L.N.; Taylor, M.S. Regioselective, borinic acid-catalyzed monoacylation, sulfonylation and alkylation of diols and carbohydrates: Expansion of substrate scope and mechanistic studies. *J. Am. Chem. Soc.* **2012**, *134*, 8260–8267.
3. Ciuffreda, P.; Alessandrini, L.; Terraneo, G.; Santaniello, E. Lipase-catalyzed selective benzoylation of 1,2-diols with vinyl benzoate in organic solvents. *Tetrahedron Asymmetry* **2003**, *14*, 3197–3201.
4. Uzawa, H.; Nishida, Y.; Ohrui, H.; Meguro, H. Application of the dibenzoate chirality method to determine the absolute configuration of glycerols and related acyclic alcohols. *J. Org. Chem.* **1990**, *55*, 116–122.
5. Prasada, A.K.; Kumara, V.; Malhotraa, S.; Ravikumarb, V.T.; Sanghvib, Y.S.; Parmar, V.S. Green'methodology for efficient and selective benzoylation of nucleosides using benzoyl cyanide in an ionic liquid. *Bioorg. Med. Chem.* **2005**, *13*, 4467–4472.
6. Babakhanov, R.A.; Zeinalov, S.B.; Sharifova, S.K.; Mekhtiev, M.S.; Agaeva, E.A. Synthesis of hydroxyamino-substituted aromatic acid esters from their chlorohydrin derivatives. *Russ. J. Org. Chem.* **1993**, *29*, 559–564.
7. Cambie, R.C.; Hayward, R.C.; Jurlina, J.L.; Rutledge, P.S.; Woodgate, P.D. reinvestigation of the Prévost reaction with N-allylmorpholine. *Aust. J. Chem.* **1981**, *34*, 1349–1351.
8. Adams, A.M.; Bois, J.D. Organocatalytic C–H hydroxylation with Oxone® enabled by an aqueous fluoroalcohol solvent system. *Chem. Sci.* **2014**, *5*, 656–659.
9. Stepanov, F.N.; Krasutskii, P.A.; Yurchenko, A.G. Interaction of 3,7-dimethylenebicyclo[3.3.1]nonane with perbenzoic and monoperphthalic acids. *Zhurnal Org. Khimii.* **1972**, 8, 1179–1183.
10. Burugupalli, S.; Shah, S.; vanderPeet, P.L.; Arora, S.; White, J.M.; Williams, S.J. Investigation of benzoyloximes as benzoylating reagents: Benzoyl-Oxyma as a selective benzoylating reagent. *Org. Biomol. Chem.* **2016**, *14*, 97–104.
11. Zhang, X.L.; Ren, B.; Ge, J.T.; Pei, Z.C.; Dong, H. *Tetrahedron* **2016**, *7*, 1005–1010.
12. Chéry, F.; Pillard, C.; Tatibouët, A.; Lucchi, O.D.; Rollina, P. Vinyl bis-sulfone methodology in thiosugars: Selective access to chiral thiovinyl sulfones and PSE oxathianes. *Tetrahedron* **2006**, *62*, 5141–1551.
13. Ren, B.; Wang, M.Y.; Liu, J.Y.; Ge, J.T.; Dong, H. Enhanced Basicity of Ag_2O by Coordination to Soft Anions. *ChemCatChem.* **2015**, *7*, 761–765.
14. Graziani, A.; Passacantilli, P.; Piancatelli, G.; Tani, S. A mild and efficient approach for the regioselective silyl-mediated protection–deprotection of C-4 hydroxyl group on carbohydrates. *Tetrahedron Lett.* **2001**, *42*, 3857–3860.
15. Muramatsu, W.; Takemoto, Y. Selectivity Switch in the Catalytic Functionalization of Nonprotected Carbohydrates: Selective Synthesis in the Presence of Anomeric and Structurally Similar Carbohydrates under Mild Conditions. *J. Org. Chem.* **2013**, *78*, 2336–2345.
16. Gray, I.J.; Kluger, R. Chelation-controlled regioselectivity in the lanthanum-promoted monobenzoylation of monosaccharides in water. *Carbohydr. Res.* **2007**, *342*, 1998–2002.
